# Comparative Studies of Different Extracts from* Eucommia ulmoides* Oliv. against Rheumatoid Arthritis in CIA Rats

**DOI:** 10.1155/2018/7379893

**Published:** 2018-07-11

**Authors:** Jian-Ying Wang, Xiao-Jun Chen, Lei Zhang, Ying-Yi Pan, Zu-Xi Gu, Shi-Min He, Zhe-Ping Song, Ying Yuan

**Affiliations:** ^1^Shanghai Innovation Center of TCM Health Service, Shanghai University of Traditional Chinese Medicine, No. 1200 Cailun Road, Pudong District, Shanghai, China; ^2^Institute of Interdisciplinary Medicine, Shanghai University of Traditional Chinese Medicine, No. 1200 Cailun Road, Pudong District, Shanghai, China; ^3^School of Pharmacy, Shanghai University of Traditional Chinese Medicine, No. 1200 Cailun Road, Pudong District, Shanghai, China; ^4^Science and Technology Experiment Center, Shanghai University of Traditional Chinese Medicine, No. 1200 Cailun Road, Pudong District, Shanghai, China; ^5^School of Basic Medical, Shanghai University of Traditional Chinese Medicine, No. 1200 Cailun Road, Pudong District, Shanghai, China

## Abstract

To compare efficacy of different extracts from* Eucommia ulmoides *Oliv. with both immune inflammation and joint destruction in collagen induced arthritis (CIA) rat model. Rats were divided into normal group (Nor), control group (CIA), TG group (treated with tripterygium glycoside), E70 group (treated with 70% ethanol extract from* Eucommia ulmoides *Oliv.), EA group (treated with ethyl acetate fraction from E70), and EN group (treated with n-butyl alcohol fraction from E70). All extracts from* Eucommia ulmoides *Oliv. could significantly inhibit ankle swelling, pathological manifestations, and cytokine levels in serum and spleen, by using foot volume measurement, H&E staining, ELISA, and RT-QPCR methods, respectively. All extracts could significantly inhibit rough joint surface and marginal osteophytes, improve RANKL/OPG ratio, and decrease MMP-9 expression, by using micro-CT and immunohistochemical staining. The activation of IKK/NF-*κ*B signaling pathway was also inhibited by all extracts. In addition, ethyl acetate fraction from E70 presented better effect on RANKL/OPG system. This study identified effective extracts from* Eucommia ulmoides *Oliv. relieving immune inflammation and maintaining structural integrity of joints in CIA rats.

## 1. Introduction

Rheumatoid arthritis (RA) is a chronic, progressive, inflammatory, and erosive autoimmune disease, of which pathological manifestations mainly include synovial inflammation and bone destruction [1]. RA has higher disability rate associated with bone loss, permanent joint damage, and eventually deformity without timely and reasonable therapy [2]. RA is a common disease globally and classified as one of the most intractable diseases by World Health Organization (WHO), because RA cannot be easily cured and can progress to lifelong illness [3]. Conventional drugs often meet limited and diminishing efficacy, as well as various perplexities due to side effects by long-term use. Traditional Chinese medicine (TCM) has good curative efficacy and safety for long-term therapy with few side effects; thus, TCM is worth further exploration for treating RA [4].

*Eucommia ulmoides *Oliv. has been used in TCM with a long history of two thousand years, which was documented in the oldest materia medica book, “Shen Nong's Herbal Classic”. It has good safety and can be processed into healthcare food certified by China's Ministry of Health. Toxicological studies [5, 6] reported that* Eucommia ulmoides *Oliv. is safe and nontoxic.* Eucommia ulmoides *Oliv. has been considered as top grade herb with multiple functions of strengthening bone, tonifying liver, and kidney [7]. Studies of modern pharmacology and molecular biology verified that crude extracts, total glycosides [8], and total lignans [7] isolated from* Eucommia ulmoides *Oliv. could safely and effectively treat bone diseases, such as osteoporosis or fractures. Additionally, the effective ingredients in* Eucommia ulmoides *Oliv. have the functional effects [5] of lowering blood pressure [9], improving immune function, antiaging, preventing inflammation, inhibiting tumor, and easing urine output. It was also found that* Eucommia ulmoides *Oliv. was one of the most frequent components in TCM prescriptions used in RA clinical therapy. Wang [10] summarized that* Eucommia ulmoides *Oliv. ranked top 5 of use frequency in 249 components by using analysis of core drugs and drug pairs in the published TCM decoction for RA clinical therapy, which suggested that* Eucommia ulmoides *Oliv. had the potential effect treating RA according to prescription studies. Furthermore, our previous study showed that ethanol extract from* Eucommia ulmoides *Oliv. could ameliorate RA in rodent model [11]. However, further identification of active fractions from* Eucommia ulmoides *Oliv and related mechanisms against RA still needed deep exploration.

In this study, the further extractive fractions from ethanol extract of* Eucommia ulmoides *Oliv. estimated the protective effect against bone destruction and inflammation and explored the preliminary mechanism. It is intended to provide scientific evidences for the systematic development of plant resources of* Eucommia ulmoides *Oliv. in medicine application.

## 2. Materials and Methods

### 2.1. Animal Care and Use Statement

Clean-grade male Wistar rats at a weight of 200±10 g were purchased from and approved by Shanghai SLAC Laboratory Animal Co., Ltd. (Animal Certificate of Conformity: SCXK (Shanghai) 2016-0002). The rats were fed adaptively for 3 days in Laboratory Animal Center of Shanghai University of Traditional Chinese Medicine. All animal experiments were performed under protocols in accordance with institutional guidelines for the care and use of laboratory animals and related ethical regulations of Shanghai University of Traditional Chinese Medicine (2016030006).

### 2.2. Collagen-II Induced Arthritis (CIA) Model and Administration

Wistar rats were induced by CIA according to the method described previously [11], and 8 rats were used as normal control (Nor). Briefly, on day 1 and day 7, rats were intradermally given primary and booster injection at the base of the tail, respectively, with 100 *μ*l solution containing 200 *μ*g bovine collagen II. A little swelling could be observed in the hind feet of CIA rats after day 10 and that became more obvious since day 14, indicating the success of CIA.

Since day 14, 40 CIA rats were randomly selected and grouped into 5 groups, with 8 rats in each group: CIA group (distilled water treatment), E70 group (4g/kg•d^−1^ 70% ethanol extract from* Eucommia ulmoides *Oliv., named as E70), EA group (4g/kg•d^−1^ ethyl acetate fraction from E70, named as EA), EN group (4g/kg•d^−1^ n-butyl alcohol fraction from E70, named as EN), and TG group (6mg/kg•d^−1^ tripterygium glycoside). CIA rats were given gavage once a day for 4 weeks. CIA group and normal group were given to distilled water in the same volume with rats in treatment group.

### 2.3. Rat Foot Swelling and Sample Collection

All rats were monitored for hindpaw swelling and body weight once a week, at day 0, before treatment, and between days 14 and 42. Hindpaw swelling measured paw volume using a paw volume meter (Shandong Academy of Medical Sciences, Shandong, China). One hour after administration in day 42, rats were euthanized with overdose of 2.5% isoflurane and sacrificed. Blood samples were collected from carotid artery. Spleens were also incised for further analysis. Ankle joint and hindpaw were removed from perfused rats with ice-cold PBS and 4% paraformaldehyde and then were fixed in 10% formalin for 4 days.

### 2.4. Micro-CT Analysis

Paraformaldehyde-fixed hindpaws were separately placed in 70% ethanol for 1 day and scanned with SkyScan-1076 (Bruker, Kontich, Belgium) to quantify bone erosion within the paws at 36.5 *μ*m isotropic voxel size, with 566 projections. Then 3D bone images of hind paws were reconstructed.

### 2.5. Histopathological Examination of the Ankle Joint

The ankle joints were decalcified in 0.5 M EDTA (pH 7.2) for 4 weeks and then embedded in paraffin and sectioned to 6 *μ*m thickness. Hematoxylin and eosin (H&E) staining and immunohistochemical staining were performed using the sections. Pathological changes were assessed by microscope.

### 2.6. Scores for Synovitis, Pannus Formation, Degradation of Cartilage, and Bone

H&E staining slides was scored by three independent observers blindly to the assay design. Cellular infiltration was scored as 0 to 3: 0, no infiltration of inflammatory cells; 1, mild infiltration; 2, moderate infiltration; and 3, severe infiltration. Pannus formation was scored as 0 to 3: 0, no pannus; 1, mild pannus; 2, moderate pannus; and 3, severe pannus. Degradation score of cartilage and bone was from 0 to 3: 0, no degradation of cartilage and bone; 1, mild degradation without narrow joint-space; 2, moderate degradation causing moderate joint-space narrowing; 3, severe degradation causing joint-space narrowing or merging.

### 2.7. Immunohistochemical Staining

Paraffin sections (6 *μ*m) of ankle joints were mounted on poly-L-lysine-coated slides, followed by dewaxing and blocking. Sections were incubated with specific antibodies for rat RANKL (1:100), OPG (1:50), and MMP-9 (1:100), prior to secondary antibody incubation. Then sections were stained with DAB, followed by hematoxylin staining for cell nucleus. The quantitative analysis for positively stained cells was performed by Image-Pro Plus software (v6.0) to express results as average optical density (AOD) at a magnification of ×200.

### 2.8. RNA Isolation and Quantitative PCR

Total RNA was isolated from rat spleen with PureLink RNA Mini Kit (Ambion) and generated cDNA using SuperScript™III First-Strand Synthesis SuperMix kit (Invitrogen) as recommended by the manufacturers. Real-time ***quantitative*** PCR was performed on cDNA by using SYBR GreenER™ qPCR SuperMix Universal kit (Invitrogen). Data were analyzed through 2^ΔΔCT^-method and normalization to GAPDH as an endogenous reference. The employed primers included IL-6, 5′- CACAGAGGATACCACCCACA -3′ and 5′- CAGAATTGCCATTGCACAAC -3′; IL-17, 5′- GCCGAGGCCAATAACTTTCT -3′ and 5′- GAGTCCAGGGTGAAGTGGAA -3′; TNF-*α*, 5′- GGAAAGCATGATCCGAGATG -3′ and 5′- CGAGCAGGAATGAGAAGAGG -3′; GAPDH, 5′- CCACCCATGGCAAATTCCATGGCA -3′ and 5′- TCTAGACGGCAGGTCAGGTCCACC -3′.

### 2.9. ELISA for Serum Levels of IL-1*β* and TNF-*α*

Enzo ELISA kits (31.3 pg/ml of minimum limit) were used to quantify serum levels of IL-1*β* and TNF-*α* according to the manufacturer's protocol. Optical density was quantified at 450 nm using a microplate reader. Concentrations were calculated according to the standard curve.

### 2.10. Western Blotting

Rat cartilages of joint were homogenized in RIPA buffer to extract total protein. Total protein determined concentration with BCA method and loaded SDS by boiling for 10 min with loading buffer, followed by separation by 10% SDS-PAGE and transferring to PVDF membrane (Pall, USA). After blocking with 5% dried milk and incubation with primary antibody at 4°C overnight, the membrane was probed with appropriate IRDye 800CW-conjugated secondary antibody at room temperature for 1 h. The protein bands determined relative quantity by image analysis system (Odyssey 3.0 software).

### 2.11. Statistical Analysis

Data was analyzed using one-way analysis of variance followed by post hoc test using the Student–Newman–Keuls (S-N-K) test for group difference if data met normal distribution and variances homogeneity. Data was analyzed using nonparametric test if data did not meet normal distribution nor variances homogeneity. Data was expressed by mean ± standard deviation and considered P<0.05 as a difference.

## 3. Results

### 3.1. Effects of Various Extracts against RA Progression and Pathology

**Figure 1:**
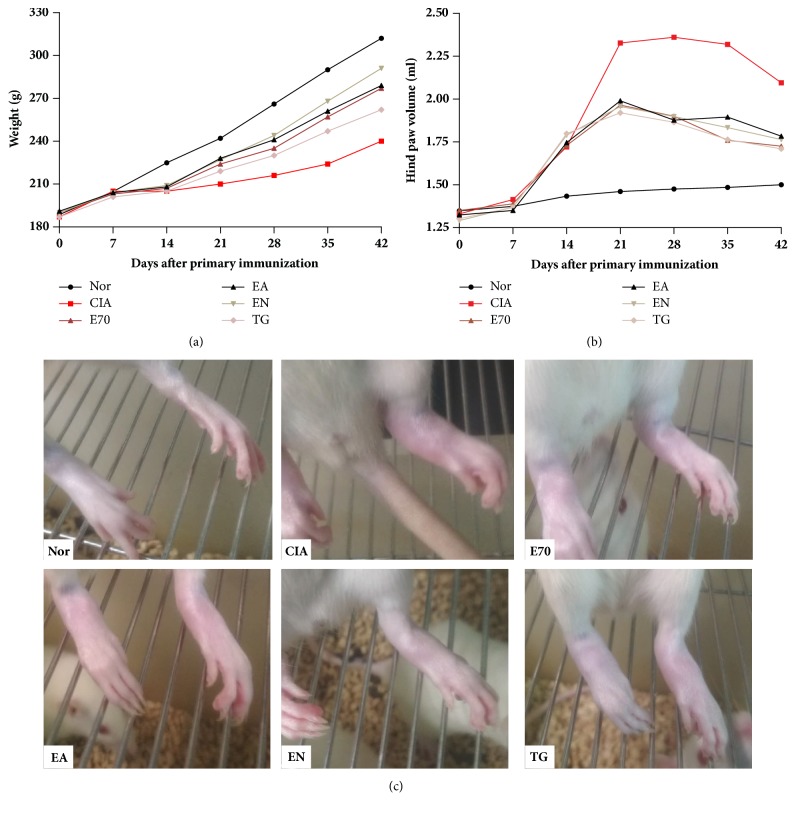
**Antiarthritic effect by extracts from* Eucommia ulmoides *Oliv. in collagen-II induced arthritis (CIA) rats.** (a) Effect by extracts on the body weight change of CIA rats. (b) Effect by extracts on ameliorating hind paw swelling of CIA rats. (c) Swelling manifestations in CIA rats after various treatments. Except normal group (Nor), CIA rats were treated daily with E70, EA, EN at 4g/kg•d^−1^, TG at 6mg/kg•d^−1^, or distilled water (CIA group) from day 14 after arthritis induction.

**Figure 2:**
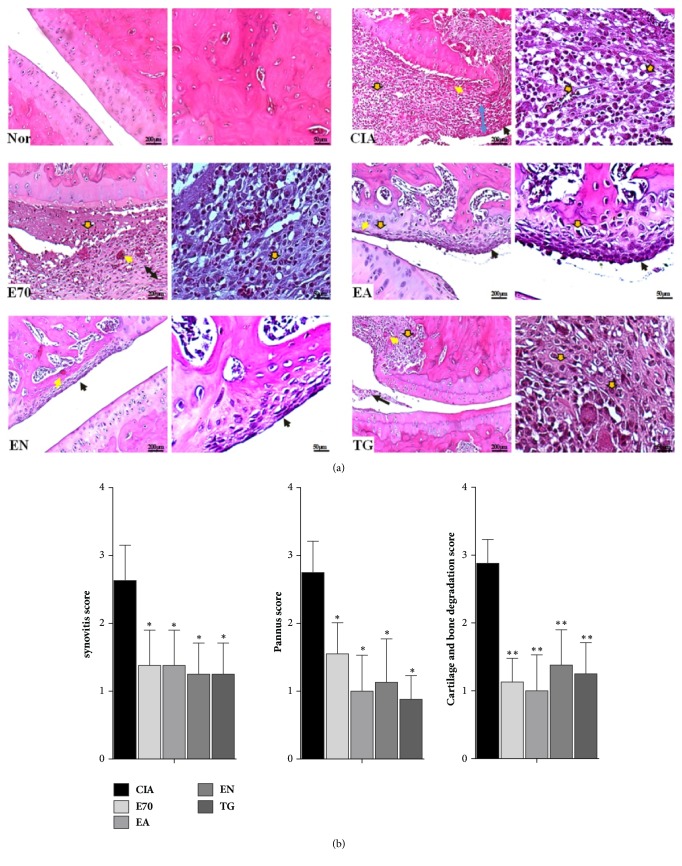
**Photomicrographs of pathological lesions of ankle joints of treated and untreated rats.** (a) H&E staining of ankle joints of CIA rats treated with various extracts. Blue arrow, joint-space; brown arrow with black border, inflammatory cell infiltration; black arrow, synovial hyperplasia; yellow arrow, pannus. (b) Pathological scores of synovitis, pannus formation, degradation of cartilage, and bone were evaluated blindly. *∗*p < 0.05 and *∗∗*p < 0.01 compared with CIA group.

Since day 10, compared to normal group, CIA rats exhibited mental weakness, sluggish activity, slight hair loss, slower weight gain (Figure 1(a)), and more serious paw swelling (Figure 1(b)). Compared to CIA group, body weights increased and paw volumes decreased significantly after drug treatment from day 21 to day 42. On day 42, there was no significant difference on body weight or paw volumes between E70, EA, and EN group (P>0.05), although body weight of EN group was shown to be a slightly heavier than those of E70 or EA groups (Figure 1(a)). Similar to paw volume result, CIA rats presented distinguishable manifestation of edema, suggesting inflammatory articular lesions (Figure 1(c)). As expectation, the photos (Figure 1(c)) of E70, EA, EN, and TG group straightly demonstrated effects of mitigating edema. Histological results (Figure 2(a)) showed that the ankle joints in CIA rats exhibited apparent inflammatory cell infiltration in soft tissue and synovial lining layer. It also showed cartilage destruction leading to pannus formation and narrowed joint-space. Treatment with TG or various extracts from* Eucommia ulmoides *Oliv. could restrict the histological development of CIA (P<0.05). EA or EN treatment remarkably reduced the synovial hyperplasia, inflammatory cell infiltration, and pannus formation compared to CIA rats (Figure 2(a)) and furthermore showed more significant improvement than E70. E70 treatment reduced the synovial hyperplasia and immune cell infiltration but still had a few pannus although it had been improved better than CIA group. According to pathological scores (Figure 2(b)) of synovitis, pannus formation, degradation of cartilage, and bone, there was no significant difference between E70, EA, and EN group (P>0.05), but all three groups present significant improvement compared to CIA group (P<0.05).

### 3.2. Effect of Various Extracts against Bone Destruction

**Figure 3:**
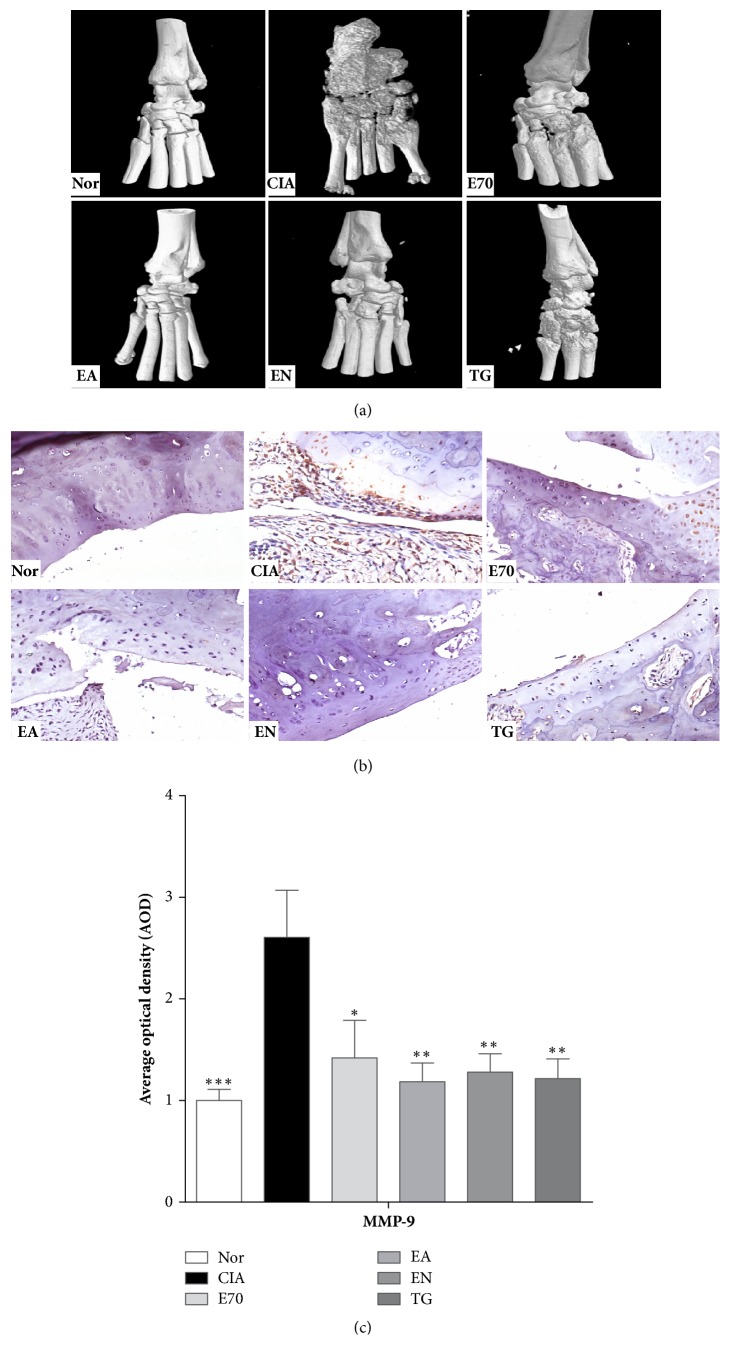
**Efficacy of extracts from* Eucommia ulmoides *Oliv. against destruction on ankle joints of CIA rats.** (a) Representative micro-CT images obtained from ankle joints of several groups. (b) Immunohistochemical detection of MMP-9 of ankle joints. (c) AOD values of MMP-9 were analyzed using Image-Pro Plus software. *∗*p < 0.05, *∗∗*p < 0.01, and *∗∗∗*p < 0.001 compared with CIA group.

Micro-CT imaging results (Figure 3(a)) showed remarkably diffuse osteogenesis and severe bone erosion with crater-shaped pits on the ankle joint of CIA group. Bone erosion was significantly improved by various extracts treatment, even less bone erosion than that of group TG (P<0.05). Bone surfaces of group EA or EN were smoother than that of group E70 (P<0.05), although there was similar improvement between groups EA and EN (P>0.05).

High expression of MMP-9 is considered as a biomarker of local inflammation of joints and is related to bone destruction. The immunohistochemical results (Figures 3(b) and 3(c)) showed high expression of MMP-9 in CIA group, distributing in synovial tissues, compared to normal group (P<0.05). The number of immune-positive cells decreased significantly after various extracts treatment. And there was no significant difference between EA and EN groups (P>0.05), of which improvements were better than that in E70 group (P<0.05).

### 3.3. Effect of Various Extracts against RANKL and OPG Expression

**Figure 4:**
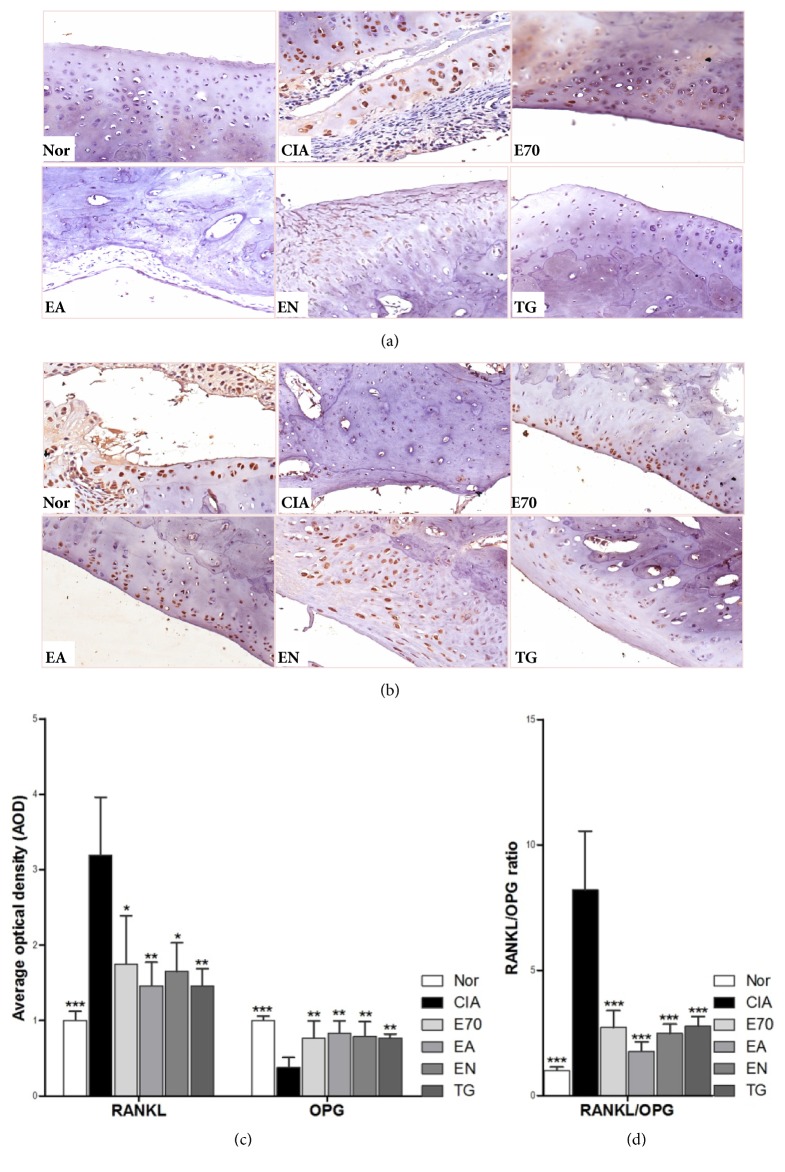
**Efficacy of extracts from* Eucommia ulmoides *Oliv. on the balance of RANKL and OPG. **Representative immunohistochemical staining obtained from ankle joints of the several groups, (a) on RANKL detection and (b) on OPG detection. (c) AOD value of RANKL or OPG was analyzed using Image-Pro Plus software. (d) The relative RANKL/OPG ratio was calculated based on AOD values of RANKL and OPG. *∗*p < 0.05, *∗∗*p < 0.01, and *∗∗∗*p < 0.001 compared with CIA group.

As shown in Figures 4(a) and 4(c), RANKL level of joint tissue in CIA group was significantly higher than that in normal group. Decrease of RANKL was the most obvious in group EA, followed by decrease by EN treatment. Compared to the average optical density (AOD) in CIA group, AOD of E70 group decreased significantly, whereas AOD was slightly higher than those in group EA and EN. As shown in Figures 4(b) and 4(c), OPG level was significantly lower in CIA group than in normal group; however, OPG increased higher in groups of E70, EA, and EN than in CIA group (P<0.05). But there was no significant difference between three treatments (P>0.05). RANKL AOD is divided from OPG AOD to obtain RANKL/OPG ratio (Figure 4(d)). EA treatment could significantly reduce CIA-induced RANKL/OPG ratio, followed by EN treatment, and E70 treatment could also reduce the ratio (P<0.05).

### 3.4. Effect of Various Extracts on the Expression and Secretion of Inflammatory Cytokines

**Figure 5:**
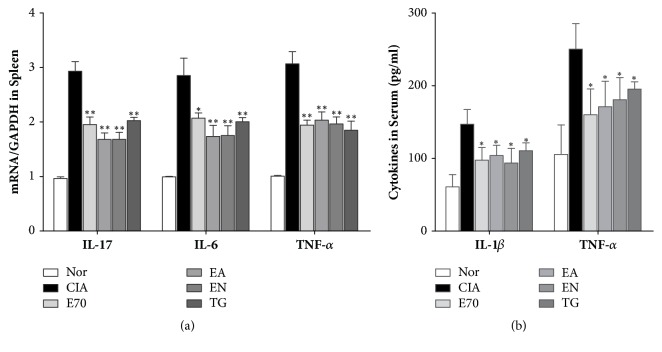
**Efficacy of extracts from* Eucommia ulmoides *Oliv. on inflammatory cytokines expression in spleen and serum.** (a) The mRNA expressions of IL-17, IL-6, and TNF-*α* in spleen were determined by real-time PCR. And the fold induction was calculated. (b) The concentrations of IL-1*β* and TNF-*α* in serum were determined by ELISA. *∗*p < 0.05 and *∗∗*p < 0.01 compared with CIA group.

As shown in Figure 5, mRNA levels of IL-6, IL-17, TNF-*α* in spleen and secretion concentrations of IL-1*β* and TNF-*α* in serum in CIA group were significantly higher than those in normal group (P<0.05). After treatment with various extracts or TG, these cytokine expressions were reduced with statistical significance (P<0.05). Through a further comparison between cytokine expressions by various extracts treatment, mRNA levels of IL-6 and IL-17 in EA or EN group were reduced more obviously than the decrease by E70 treatment (P <0.05), whereas improved effect of TNF-*α* was the same in three groups (P > 0.05).

### 3.5. Effect of Various Extracts on Molecules in NF-*κ*B Pathway

**Figure 6:**
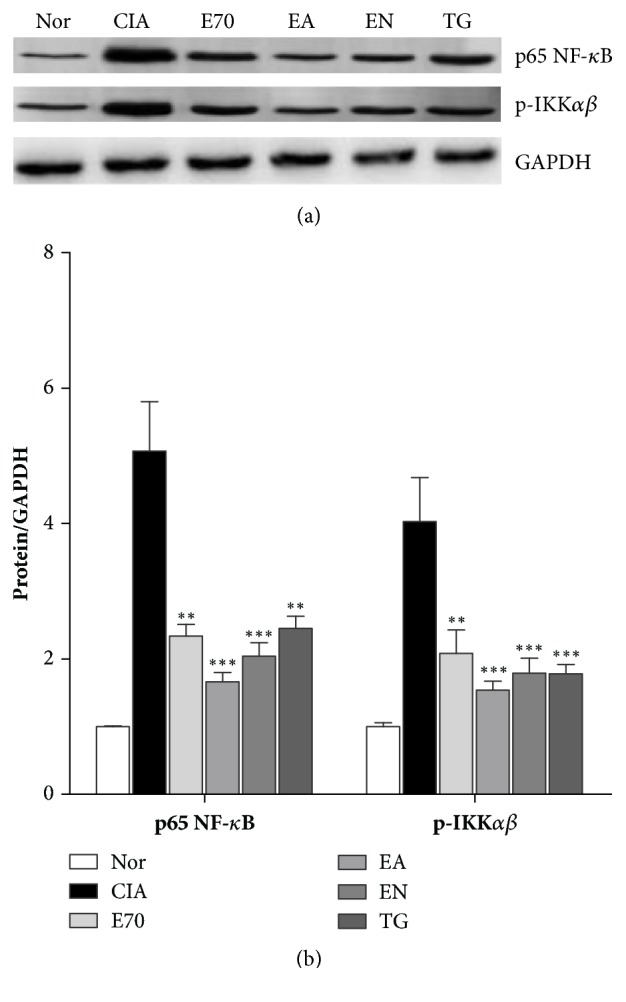
**Efficacy of extracts from* Eucommia ulmoides *Oliv. on NF-*κ*B signaling pathway. **(a) Representative expression for p65 NF-*κ*B and p-IKK*αβ* obtained from ankle joints of the several groups detected by western blotting. (b) Densitometric quantification of p65 NF-*κ*B and p-IKK*αβ* in several groups with GAPDH as loading control. *∗∗*p < 0.01 and *∗∗∗*p < 0.001 compared with CIA group.

As shown in Figure 6, after collagen II injection, the protein expressions of p65NF-*κ*B and p-IKK*αβ* of joint tissues in CIA group were significantly higher than those in normal group (P<0.05). EA significantly decreased the elevated expression of p65NF-*κ*B and p-IKK*αβ* in CIA rats (P<0.05), with better improvement than EN-treated CIA rats (P<0.05). Moreover, EN treatment decreased p65NF-*κ*B and p-IKK*αβ* much more than E70-treated CIA rats (P<0.05).

## 4. Discussion

RA performs insidious onset and gradual advances [12]. Initially, RA affects many organs and tissues outside joints and performs varied symptom, such as fatigue, general discomfort, weight loss and low fever sustaining for a few weeks, or high fever in some patients. Then, RA patients gradually show typical joint symptoms [13]. Joint symptoms can be divided into synovial inflammation and joint structure destruction, which present certain reversibility after therapy [14] and become gradually difficult to reverse once bone destruction appears [15], respectively. Because etiology and pathogenesis of RA are not completely understood, it still lacks effectively medical treatment to prevent and cure RA. It is the therapeutic goal to find effective and safe agents that can reduce joint symptoms, prevent and reduce joint damage, protect joint function, and delay RA progression with maximum extent [16].* Eucommia ulmoides* Oliv. is a valuable and safe TCM medicine and has a wide range of clinical application, especially in strengthening bone and improving bone metabolism [7]. Patent medicines or TCM prescriptions with* Eucommia ulmoides* Oliv. as principal ingredient have been extensively developed and widely used in clinic and have produced good curative effects. In addition,* Eucommia ulmoides* Oliv. has also effects of anti-inflammation, activation of blood circulation, analgesia, and so on, to improve the human body [9]. According to functions and characteristics of* Eucommia ulmoides* Oliv., in this present study, the effects of* Eucommia ulmoides* Oliv. against inflammation and joint destruction were investigated, and the preliminary mechanism was explored.

Osteoclasts (OCs) play a key role in bone destruction of RA [17]. RANKL/RANK/OPG is a signaling pathway closely related to differentiation and maturation of OCs [18]. In RA pathogenesis, RANKL combines with RANK, in turn to induce differentiation and maturation of OCs. However, OPG, as the decoy receptor of RANKL, inhibits combination of RANKL and RANK, to inhibit the differentiation of OC precursor cells and the followed bone absorption activity of matured OCs, as well as inducing OCs apoptosis. RANKL/OPG ratio counts the determining balance in RANKL/RANK/OPG system. The formation, differentiation, activation, and apoptosis of OCs are actually determined by the concentration ratio of RANKL to OPG in the local microenvironment. It is suggested that* Eucommia ulmoides* Oliv. could alleviate the deteriorated RANKL/RANK/OPG system since all of extracts could improve expression of RANKL and OPG, as well as RANKL/OPG ratio in CIA rats. Although there was no significant difference of OPG AOD between three* Eucommia ulmoides* Oliv.-treated groups, RANKL expression and RANKL/OPG ratio still presented a declined trend in the order of group EA, EN, and E70, suggesting that RANKL expression and RANKL/OPG ratio are more sensitive indicators than OPG.

By conformational changes of RANK combined with RANKL, TRAF6 is integrated to intracellular region of RANK and recruits more signaling molecules, to activate IKK/NF-*κ*B signaling pathway. It indicated that RANKL/RANK/OPG system might take effect on p-IKK*αβ* expression and its downstream NF-*κ*B molecule in joint tissues, to trigger NF-*κ*B translocation into cell nucleus and the transcription of osteoclast-forming genes [19]. MMP-9 expression is positively regulated by transcriptional activity of NF-*κ*B [20], and high MMP-9 is a biomarker related to bone destruction [21]. In this present study, increased expressions of p65NF-*κ*B, p-IKK*αβ* and MMP-9 were inhibited, and bone erosion shown by micro-CT could be alleviated by various extracts from* Eucommia ulmoides* Oliv. It is speculated that p-IKK*αβ* was downregulated through RANKL/RANK/OPG and then decreased the nuclear translocation of p65NF-*κ*B and its transcriptional effect on MMP-9. These experimental results indicated that improved RANKL/OPG ratio by* Eucommia ulmoides* Oliv. might be an important balance affecting the occurrence and degree of local bone erosion in CIA rats.

In RA patients, multiple inflammatory cytokines produced by systemic and local inflammation can induce OC-mediated bone destruction. TNF-*α* induces RANKL which was produced by osteoblasts (OBs) [22] or bone marrow stromal cells (BMSCs), to promote OCs formation. TNF-*α* can also stimulate T lymphocytes [23] to produce RANKL and M-CSF and even directly induce formation of multinucleated or osteolytic cells in the presence of M-CSF. Therefore, TNF-*α* inhibition is beneficial to prevent RA. IL-1*β* is another important inflammatory cytokine increasing in body of RA patients, which leads to elevated expression of RANKL in OBs and then supports the survival, multinucleation, and activation of OC-like cells [24]. IL-6 and IL-17 are both important cytokines inducing bone resorption [25]. Both IL-6 and IL-17 induce RANKL expression, to cause joint destruction. Furthermore, IL-17, produced by activated Th17 cells, can induce secretion of various inflammatory cytokines by synovial cells, cartilage, and bone cells, leading to RA symptoms [26]. Then, these cytokines further maintain and increase Th17 cell quantity, to secret more IL-17 to participate in the above processes. The cyclical feedback amplifies the whole inflammatory cascade reaction and results in the chronic inflammatory state. Inflammatory cytokines are closely related to downstream NF-*κ*B expression and activation. IL-17 and TNF-*α* have important regulatory effect on NF-*κ*B. Combined effect of IL-17, IL-1*β* and TNF-*α* enhances NF-*κ*B expression in OC-like cells and increases IL-6 expression. IL-1*β* and TNF-*α* are considered as important inducers of NF-*κ*B and are involved in positive feedback to enhance NF-*κ*B. NF-KB activated by IL-1*β* and TNF-*α* comes back to increases gene transcription of IL-1*β* and TNF-*α*, leading to further enhancement of NF-*κ*B. In this study, elevated levels of TNF-*α* and IL-1*β* in serum and increased mRNA expressions of IL-17, IL-6, and TNF-*α* in spleen of CIA rats were found.* Eucommia ulmoides* Oliv. inhibited the increased cytokines, which proved anti-inflammatory effect of* Eucommia ulmoides* Oliv. Furthermore, it was speculated that multiple inflammatory cytokines might exert some certain inhibition to NF-*κ*B expression in the joint tissue. The principle known components of* Eucommia ulmoides *Oliv. are lignins, iridoids, flavonoids, polysaccharides, phenolics,* etc.,* and most of them are polar compounds [27]. According to He's report [5], 112 compounds have been isolated from* Eucommia ulmoides *Oliv. Among them, lignins, iridoids, and phenolics account for the major proportion, followed by flavonoids and other types of compounds. Polar compounds can be extracted using ethyl acetate or n-butanol as solvent. In this study, the therapeutic effect against RA by EA treatment was a little better than those by EN or E70, in improving RANKL/OPG ratio and decreasing protein levels of p65 NF-*κ*B and p-IKK*αβ* in joint tissue. It is presumed that this may be related to the higher concentration of effective compounds against RA by EA extraction, such as iridoids or flavonoids. Geniposide, an iridoids compound, is recognized as a key active ingredient from* Eucommia ulmoides* Oliv. and is also a candidate compound for RA treatment [28]. Wogonoside, quercetin, and its derivatives are flavonoids compounds, and quercetin [29] has proved effect of treating arthritis. The therapeutic effect by EN treatment was better than that by E70. It is presumed that EN fraction might be enriched with most of lignins and phenolics to resist RA. Lignins accounts for the largest proportion of compounds in* Eucommia ulmoides* Oliv., while it mainly reported PDE inhibitor activity for lignins [30]. Phenolics also include many antiarthritic compounds, such as caffeic acid [31], chlorogenic acid [32], syringin [33], and catechin [34]. Taken together, the synergy of various chemicals in extracts builds the merits of multiple-targets and multiple-function, which is in accordance with other TCM's characteristic, such as Bi-Qi capsule's multiple effects against RA in alleviating cartilage destruction, local inflammation, and systemic inflammation [35].

## 5. Conclusions

In summary, three different extracts from* Eucommia ulmoides* Oliv. could significantly inhibit bone destruction, synovial inflammation, and systemic inflammation. And among them, ethyl acetate fraction from 70% ethanol extract presents a little better effect in improving RANKL/OPG ratio and decreasing NF-*κ*B pathway than those by 70% ethanol extract or n-butyl alcohol fraction from 70% ethanol extract. Results of this study remind us that effective compounds of* Eucommia ulmoides* Oliv. can be further explored from these two fractions.

## Data Availability

The data used to support the findings of this study are available from the corresponding author upon request.

## References

[1] Brown J. M., Ross E., Desanti G. (2017). Detection and characterisation of bone destruction in murine rheumatoid arthritis using statistical shape models. *Medical Image Analysis*.

[2] Shim J., Stavre Z., Gravallese E. M. (2018). Bone loss in rheumatoid arthritis: basic mechanisms and clinical implications. *Calcified Tissue International*.

[3] Kiadaliri A. A., Felson D. T., Neogi T., Englund M. (2017). Brief report: rheumatoid arthritis as the underlying cause of death in thirty-one countries, 1987–2011: trend analysis of world health organization mortality database. *Arthritis & Rheumatology*.

[4] Huang M.-C., Pai F.-T., Lin C.-C. (2015). Characteristics of traditional Chinese medicine use in patients with rheumatoid arthritis in Taiwan: a nationwide population-based study. *Journal of Ethnopharmacology*.

[5] He X., Wang J., Li M. (2014). *Eucommia ulmoides* oliv.: ethnopharmacology, phytochemistry and pharmacology of an important traditional Chinese medicine. *Journal of Ethnopharmacology*.

[6] Liu Y. F., Chen J. W., Gong P. F. (2006). Studies on the subchronic toxicology of Eucommia ulmoides extract. *Lishizhen Medicine and Materia Medica Research*.

[7] Zhang R., Pan Y.-L., Hu S.-J., Kong X.-H., Juan W., Mei Q.-B. (2014). Effects of total lignans from *Eucommia ulmoides* barks prevent bone loss in vivo and in vitro. *Journal of Ethnopharmacology*.

[8] Li Y., Hu W., Han G. (2018). Involvement of bone morphogenetic protein–related pathways in the effect of aucubin on the promotion of osteoblast differentiation in MG63 cells. *Chemico-Biological Interactions*.

[9] Li Z.-Y., Gu J., Yan J. (2013). Hypertensive cardiac remodeling effects of lignan extracts from *Eucommia ulmoides* Oliv. bark—a famous traditional Chinese medicine. *American Journal of Chinese Medicine*.

[10] Wang J.-Q., Tong S.-H. (2011). Prescriptions analysis of traditional Chinese medicine for rheumatic arthralgia. *China Pharmaceuticals*.

[11] Wang J.-Y., Yuan Y., Chen X.-J. (2016). Extract from Eucommia ulmoides Oliv. ameliorates arthritis via regulation of inflammation, synoviocyte proliferation and osteoclastogenesis in vitro and in vivo. *Journal of Ethnopharmacology*.

[12] Tokoroyama T., Ando M., Setoguchi K., Tsuchiya K., Nitta K. (2017). Prevalence, incidence and prognosis of chronic kidney disease classified according to current guidelines: a large retrospective cohort study of rheumatoid arthritis patients. *Nephrology Dialysis Transplantation*.

[13] Bellucci E., Terenzi R., Paglia G. M (2016). One year in review 2016: pathogenesis of rheumatoid arthritis. *Clinical and Experimental Rheumatology*.

[14] Vandooren B., Cantaert T., Van Lierop M.-J. (2009). Melanoma inhibitory activity, a biomarker related to chondrocyte anabolism, is reversibly suppressed by proinflammatory cytokines in rheumatoid arthritis. *Annals of the Rheumatic Diseases*.

[15] Oshita K., Yamaoka K., Tanaka Y. (2013). Regulation of osteoclastogenesis by human mesenchymal stem cells leading to application of a novel treatment for rheumatoid arthritis. *Journal of UOEH*.

[16] Kaneko Y., Takeuchi T. (2014). A paradigm shift in rheumatoid arthritis over the past decade. *Internal Medicine*.

[17] Okamoto K., Takayanagi H. (2011). Osteoclasts in arthritis and Th17 cell development. *International Immunopharmacology*.

[18] Tanaka S. (2007). Signaling axis in osteoclast biology and therapeutic targeting in the RANKL/RANK/OPG system. *American Journal of Nephrology*.

[19] Li X., He L., Hu Y. (2013). Sinomenine suppresses osteoclast formation and mycobacterium tuberculosis H37Ra-induced bone loss by modulating RANKL signaling pathways. *PLoS ONE*.

[20] Zhang C., Li Y., Qian Z.-J., Lee S.-H., Li Y.-X., Kim S.-K. (2011). Dieckol from *Ecklonia cava* regulates invasion of human fibrosarcoma cells and modulates MMP-2 and MMP-9 expression via NF-*κ*B pathway. *Evidence-Based Complementary and Alternative Medicine*.

[21] Jakobs M., Häupl T., Krenn V., Guenther R. (2009). MMP- and FAP-mediated non-inflammation-related destruction of cartilage and bone in rheumatoid arthritis. *Zeitschrift für Rheumatologie*.

[22] Pathak J. L., Bravenboer N., Verschueren P. (2014). Inflammatory factors in the circulation of patients with active rheumatoid arthritis stimulate osteoclastogenesis via endogenous cytokine production by osteoblasts. *Osteoporosis International*.

[23] Huang B., Wang Q. T., Song S. S. (2012). Combined use of etanercept and MTX restores CD4+/CD8+ ratio and Tregs in spleen and thymus in collagen-induced arthritis. *Inflammation Research*.

[24] Shiozawa S., Tsumiyama K., Yoshida K., Hashiramoto A. (2011). Pathogenesis of joint destruction in rheumatoid arthritis. *Archivum Immunologiae et Therapia Experimentalis*.

[25] Bossaller L., Rothe A. (2013). Monoclonal antibody treatments for rheumatoid arthritis. *Expert Opinion on Biological Therapy*.

[26] Duvallet E., Semerano L., Assier E., Falgarone G., Boissier M.-C. (2011). Interleukin-23: A key cytokine in inflammatory diseases. *Annals of Medicine*.

[27] Ding Y., Dou D., Guo Y., Li Q. (2014). Simultaneous quantification of eleven bioactive components of male flowers of Eucommia ulmoides oliver by HPLC and their quality evaluation by chemical fingerprint analysis with hierarchical clustering analysis. *Pharmacognosy Magazine*.

[28] Deng R., Li F., Wu H. (2018). Anti-inflammatory mechanism of geniposide: inhibiting the hyperpermeability of fibroblast-like synoviocytes via the RhoA/p38MAPK/NF-*κ*B/F-actin signal pathway. *Frontiers in Pharmacology*.

[29] Yang Y., Zhang X., Xu M., Wu X., Zhao F., Zhao C. (2018). Quercetin attenuates collagen-induced arthritis by restoration of Th17/Treg balance and activation of Heme Oxygenase 1-mediated anti-inflammatory effect. *International Immunopharmacology*.

[30] Shi S.-Y., Peng M.-J., Zhang Y.-P., Peng S. (2013). Combination of preparative HPLC and HSCCC methods to separate phosphodiesterase inhibitors from *Eucommia ulmoides* bark guided by ultrafiltration-based ligand screening. *Analytical and Bioanalytical Chemistry*.

[31] Wang W., Sun W., Jin L. (2017). Caffeic acid alleviates inflammatory response in rheumatoid arthritis fibroblast-like synoviocytes by inhibiting phosphorylation of I*κ*B kinase *α*/*β* and I*κ*B*α*. *International Immunopharmacology*.

[32] Chauhan P. S., Satti N. K., Sharma P., Sharma V. K., Suri K. A., Bani S. (2012). Differential effects of chlorogenic acid on various immunological parameters relevant to rheumatoid arthritis. *Phytotherapy Research*.

[33] Song Y. Y., Li Y., Zhang H. Q. (2010). Therapeutic effect of syringin on adjuvant arthritis in rats and its mechanisms. *Yao Xue Xue Bao*.

[34] Tang L., Wei W., Wang X. (2007). Effects and mechanisms of catechin for adjuvant arthritis in rats. *Advances in Therapy*.

[35] Wang K., Zhang D., Liu Y. (2018). Traditional Chinese medicine formula Bi-Qi capsule alleviates rheumatoid arthritis-induced inflammation, synovial hyperplasia, and cartilage destruction in rats. *Arthritis Research & Therapy*.

